# Association of Chronic Condition Special Needs Plan With Hospitalization and Mortality Among Patients With End-Stage Kidney Disease

**DOI:** 10.1001/jamanetworkopen.2020.23663

**Published:** 2020-11-02

**Authors:** Bryan N. Becker, Jiacong Luo, Kathryn S. Gray, Carey Colson, Dena E. Cohen, Stephen McMurray, Bryan Gregory, Nathan Lohmeyer, Steven M. Brunelli

**Affiliations:** 1 DaVita, Inc, Denver, Colorado; 2 DaVita Clinical Research, Minneapolis, Minnesota; 3 DaVita VillageHealth, Denver, Colorado

## Abstract

**Importance:**

While several studies have demonstrated the benefit of enrollment in chronic condition special needs plans (C-SNPs) for other chronic diseases (eg, diabetes), there is no evaluation of the association of C-SNPs with outcomes among patients with end-stage kidney disease (ESKD).

**Objective:**

To examine whether and to what degree C-SNP enrollment was associated with improved clinical outcomes and quality of life in patients with ESKD.

**Design, Setting, and Participants:**

This multicenter cohort study included 2718 patients who were newly enrolled in an ESKD C-SNP between January 1, 2013, and September 30, 2017, and receiving dialysis from DaVita Kidney Care. Patients were followed up until death, loss to follow-up, or end of study (ie, December 31, 2018). Enrollees in C-SNP were matched via multiple clinical and demographic characteristics with 2 different control populations, as follows: (1) those in the same facilities (n = 2545) or (2) those in similar counties (n = 1986). Patients enrolled in CareMore C-SNPs (n = 206) were excluded from the study. Data analysis was conducted June to December 2019.

**Exposures:**

Standard ESKD care with dialysis plus access to an integrated care team who worked with the patient and the dialysis team, comprehensive health assessments done by the integrated care team, and access to select benefits (such as vision and dental care) as a C-SNP enrollee.

**Main Outcomes and Measures:**

Hospitalizations, mortality, laboratory values indicative of metabolic control, and Kidney Disease Quality of Life 36-item (KDQOL-36) survey scores.

**Results:**

The 2545 C-SNP enrollees in the facility-matched analysis had a mean (SD) age of 57.2 (12.9) years, and included 968 (38.0%) women, 1328 (52.2%) Hispanic individuals, and 553 (21.7%) African American individuals. The 1986 C-SNP enrollees in the county-matched analysis had a mean (SD) age of 57.8 (12.2) years, with 705 (35.5%) women, 1085 (54.6%) Hispanic individuals, and 472 (23.8%) African American individuals. Compared with patients not enrolled in C-SNP, enrollees had lower hospitalization rates, with incidence rate ratios of 0.90 (95% CI, 0.84-0.97; *P* = .006) in the facility-matched analysis and 0.76 (95% CI, 0.70-0.83; *P* < .001) in the county-matched analysis. Compared with patients not enrolled in C-SNP, enrollees had decreased mortality risk in the same facilities (hazard ratio, 0.77; 95% CI, 0.68-0.88; *P* < .001) and in the same counties (hazard ratio, 0.77; 95% CI, 0.66-0.88; *P* < .001). No significant differences were observed between C-SNP enrollees and matched patients in metabolic laboratory values or KDQOL-36 survey scores.

**Conclusions and Relevance:**

This cohort study found a positive association of C-SNP enrollment with lower rates of hospitalization and mortality. The findings suggest that the additional services and benefits C-SNPs provide may improve outcomes compared with standard of care for patients with ESKD.

## Key Points

**Question:**

Is enrollment in a chronic condition special needs plan (C-SNP) associated with improved outcomes in patients with end-stage kidney disease (ESKD)?

**Findings:**

In this cohort study of 2545 C-SNP enrollees matched at the facility level to patients not enrolled in C-SNP and 1986 C-SNP enrollees matched at the county level to patients not enrolled in C-SNP, C-SNP enrollees had significantly lower hospitalization rates and mortality risk than matched patients who were not enrolled in a C-SNP.

**Meaning:**

The findings of this study suggest that enrollment in a C-SNP may improve outcomes compared with standard care for patients with ESKD.

## Teaser

This cohort study examines whether enrollment in a chronic condition special needs plan is associated with improved clinical outcomes and quality of life among patients with end-stage kidney disease (ESKD).

## Introduction

Patients with end-stage kidney disease (ESKD) are logical candidates for coordinated care or disease management programs. Most have multiple chronic conditions, including heart failure, diabetes, and hypertension—conditions that can be successfully addressed via disease management programs. Clinical practice guidelines and evidence-based interventions can be used to support and manage their care. Moreover, they either receive treatment 3 times per week or have a regular evaluation at the dialysis clinic if being treated someplace else, such as at home. In addition, they often have a network of clinicians involved in their care to treat their multiple comorbidities.

Coordinated care programs for patients with ESKD evolved in the 1990s.^1^ Mortality rates and clinical parameters improved among patients in these programs compared with patients receiving general care, although hospitalizations were not significantly different between patient groups.^2^ Additional analyses suggested that a different care model, with a care manager, a quality management system, and differential physician engagement, reduced mortality and hospitalization.^2^ Based on these data, the Centers for Medicare & Medicaid Services (CMS) contracted with Medicare Advantage in a disease management demonstration to determine whether plan-based interventions and resources could improve clinical outcomes and reduce costs for patients with ESKD.^3^ Patient survival improved and cardiovascular-related hospitalizations declined compared with patients in the fee-for-service program during the demonstration’s first 3 years. The demonstration also highlighted the benefits of ESKD-specific treatments, such as nutritional supplementation.

CMS subsequently included ESKD in the chronic or disabling conditions eligible for enrollment in chronic condition special needs plans (C-SNPs). C-SNPs are a type of Medicare Advantage offering targeted or specialized services for Medicare beneficiaries who have a severe or disabling chronic condition. The plan receives payments per member per month and is at risk for coordinating and managing the care of the individuals enrolled in the plan. The plan may also provide specific benefits and health care networks for the beneficiaries enrolled. These vary based on the plan and may include access to preventive vision and dental services, nutrition support, and access to transportation, among other options that are determined by the plan. Special needs plans also have to meet certain requirements, such as maintaining a model of care that outlines the plan’s care coordination, care management, and quality strategies. Regulations require that CMS approves the model of care but do not specify the interventions to be included. In light of the theoretical and extrapolated benefits of C-SNPs, we examined whether C-SNP enrollment was associated with improved clinical outcomes and quality of life in a real-world setting among ESKD patients who met eligibility criteria.

## Methods

### Study Population

Data on the study population, including patient characteristics and outcome data, were obtained from DaVita Kidney Care internal data. Patients were eligible for inclusion in this study if they were newly enrolled in an ESKD C-SNP between January 1, 2013, and September 30, 2017, and were receiving dialysis from DaVita Kidney Care; the index date for each C-SNP enrollee was defined as the first day of the third month after C-SNP enrollment, thereby allowing for a latency period during which the association of C-SNP enrollment with outcomes might commence. As of the index date, C-SNP enrollees had to meet the following criteria to be included in this study: aged at least 18 years and with no commercial secondary insurance. C-SNP enrollees were matched with patients who, as of the index date of the corresponding case, were not enrolled in a C-SNP, had Medicare (Part A or B) as primary insurance, and did not have commercial secondary insurance.

Patients were excluded if ever enrolled in a CareMore C-SNP. There were 206 patients with evidence of previous enrollment in CareMore C-SNPs (46 C-SNP enrollees; 160 otherwise-eligible matched patients). Because CareMore C-SNPs provided a different care model for patients, with different interventions and a different care team structure, there was concern regarding how to handle this patient population while acknowledging the success of CareMore C-SNPs in general.^4^ Thus, to avoid confounding, patients with a CareMore C-SNP treatment history were excluded from both study groups. Patients who received benefits through the Department of Veterans Affairs were also excluded from the analyses because of contractual stipulations.

Because this study was conducted using deidentified patient data, according to Title 45, part 46 of the US Department of Health and Human Services’ Code of Federal Regulations, it was deemed exempt from institutional review board or ethics committee approval. We adhered to the Declaration of Helsinki,^5^ and informed consent was not required. This study followed the Strengthening the Reporting of Observational Studies in Epidemiology (STROBE) reporting guideline.

### Control Eligibility and Matching

Two different matching strategies were used to address potential confounding: facility-level and county-level matching. In the facility-level analysis, matched patients for each C-SNP enrollee were those patients who, as of the C-SNP enrollee’s index date, were receiving dialysis at the same facility and met all other inclusion criteria but had not enrolled in a C-SNP. Patients were matched to C-SNP enrollees based on dialysis facility; index month; modality (ie, hemodialysis or peritoneal dialysis; exact matches); propensity score that considered clinical and demographic variables, including age, sex, race and ethnicity, employment status, body mass index (BMI; calculated as weight in kilograms divided by height in meters squared), ESKD etiology, dialysis vintage, transplant history, Charlson Comorbidity Index score, and comorbid diagnoses of diabetes, congestive heart failure, chronic obstructive pulmonary disease, peripheral vascular disease, atrial fibrillation, HIV/AIDS, depression, hepatitis B or C, gastrointestinal bleeding, liver disease, lupus, neurological disorders, or ischemic heart disease.

In the county-level analysis, matched patients were those who, as of the C-SNP enrollee’s index date, received dialysis in a similar county where no C-SNP was available and met all other inclusion criteria. Similar counties were identified using the k-means clustering technique,^6^ performed prior to patient-level matching. The input variables used to generate clusters of similar counties included such factors as county population, percentage of population younger than 18 years, percentage of population with English proficiency, percentage of population who are non-Hispanic African American individuals, number of primary care physicians, number of uninsured adults, child mortality rate, number of children in poverty, physical inactivity of residents, residents with poor or fair health, and proportion of population who drives alone to work. Additional details about DaVita, CareMore, and control matching are provided in the eAppendix in the Supplement.

Eligible matched patients were matched 1:1 with C-SNP enrollees based on county cluster, index month, sex, race, etiology, modality (ie, hemodialysis or peritoneal dialysis; exact matches) and a propensity score (eFigure in the Supplement) that considered the aforementioned clinical and demographic variables.

### Follow-up and Outcomes

Patients were followed up until death, loss to follow-up (including for transplantation, transfer of care, kidney function recovery, withdrawal from dialysis, and C-SNP termination for study patients or change in insurance status for matched patients, such as to violate the eligibility criteria), or until the end of study (December 31, 2018). Patient data were censored for death or loss to follow-up (eTable 1 in the Supplement). Outcomes were assessed from index date until censoring or study end, whichever occurred first. Assessed outcomes include hospitalizations, mortality, laboratory values indicative of metabolic control (ie, serum calcium, phosphate, potassium, parathyroid hormone levels; each evaluated as the mean during follow-up for each patient), and Kidney Disease Quality of Life 36-item (KDQOL-36) survey scores (evaluated as the mean for each component score for each patient when multiple surveys, which are typically administered annually, were present).

### Statistical Analysis

Patient characteristics were summarized as of index month. Balance within each matched cohort was assessed using standardized differences. Within each matched cohort, outcomes were compared across exposure categories using general and generalized linear models with distributions dictated by data type (ie, hospitalization, metabolic indices, KDQOL-36 scores) or Fine and Gray subdistribution hazard models (for mortality). Data for hospitalizations and mortality are reported as incident rate ratios and hazard ratios (HRs), respectively, with 95% CIs; further adjustment of models for these 2 outcomes by the factors used in matching (represented by the propensity score) did not affect the reported point estimates and confidence intervals. Data for laboratory values are reported as means with 95% CIs. Data for KDQOL-36 survey values are reported as component scores with minimum, 25th percentile, median, 75th percentile, and maximum for each. Data analysis was conducted in SAS version 9.4 (SAS Institute). A *P* ≤ .05 was considered significant. KDQOL-36 scores between groups were compared using a mixed model to account for multiple surveys contributed by individual patients. The *P* value represents the Type III test of fixed effects for the binary indicator for SNP enrollment in that model.

## Results

### Baseline Characteristics

Overall, 2718 C-SNP enrollees met inclusion criteria. Of these, 2545 patients were successfully matched to patients in the facility-matched analysis. Baseline characteristics of the C-SNP and control groups were similar in terms of age (mean [SD] age, 57.2 [12.9] years vs 57.1 [13.0] years), sex (968 [38.0%] women vs 997 [39.2%] women), distribution of race (553 [21.7%] African American vs 537 [21.1%] African American) and ethnicity (1328 [52.2%] Hispanic vs 1352 [53.1%] Hispanic), BMI (mean [SD], 27.7 [6.8] vs 27/7 [7.0]), etiology of kidney disease (eg, diabetes: 1245 [48.9%] vs 1227 [48.2%]), and vintage (mean [SD], 46.3 [39.7] months vs 48.0 [41.3] months) (standardized difference <10% for all characteristics) (Table 1). Characteristics of unmatched patients are summarized in eTable 2 in the Supplement. Notably, the study population had more than 50% Hispanic individuals, and Hispanic and African American individuals accounted for nearly 75% of the study population. The ESKD etiology reflected the distribution in data from the United States Renal Data System.^7^

**Table 1.** Characteristics of C-SNP Enrollees and Matched Patients Across Same Facility and Similar County Analyses

Characteristic	Patients in same facility, No. (%)			Patients in similar counties, No. (%)		
	Matched (n = 2545)	C-SNP (n = 2545)	Standardized difference, %	Matched (n = 1986)	C-SNP (n = 1986)	Standardized difference, %
Age, mean (SD), y	57.1 (13.0)	57.2 (12.9)	0.7	58.1 (12.2)	57.8 (12.2)	−2.2
Women	997 (39.2)	968 (38.0)	−2.3	705 (35.5)	705 (35.5)	0^a^
Race/ethnicity						
White	399 (15.7)	404 (15.9)	6.9	277 (14.0)	277 (14.0)	0^a^
Black	537 (21.1)	553 (21.7)		472 (23.8)	472 (23.8)	
Hispanic	1352 (53.1)	1328 (52.2)		1085 (54.6)	1085 (54.6)	
Asian	147 (5.8)	133 (5.2)		75 (3.8)	75 (3.8)	
Other or missing	110 (4.3)	127 (5.0)		77 (3.9)	77 (3.9)	
BMI, mean (SD)	27.7 (7.0)	27.7 (6.8)	1.2	28.0 (6.8)	27.9 (6.6)	−0.9
Etiology						
Diabetes	1227 (48.2)	1245 (48.9)	3.2	1107 (55.7)	1107 (55.7)	0^a^
Hypertension	736 (28.9)	735 (28.9)		579 (29.2)	579 (29.2)	
Other	582 (22.9)	565 (22.2)		300 (15.1)	300 (15.1)	
Vintage, mean (SD), mo	48.0 (41.3)	46.3 (39.7)	−4.3	45.1 (37.1)	44.2 (37.7)	−2.4
Modality						
HHD	<10	<10	NA	0	0	0^a^
ICHD	2360 (92.7)	2360 (92.7)		1855 (93.4)	1855 (93.4)	
NOC	NS	NS		0	0	
PD	166 (6.5)	166 (6.5)		131 (6.6)	131 (6.6)	
CCI, mean (SD)	5.1 (1.7)	5.1 (1.7)	−1.2	5.2 (1.6)	5.2 (1.5)	−1.2
Diabetes	2042 (80.2)	2022 (79.5)	−2.0	1631 (82.1)	1661 (83.6)	4.0
CHF	254 (10.0)	249 (9.8)	−0.7	194 (9.8)	184 (9.3)	−1.7
COPD	60 (2.4)	63 (2.5)	0.8	44 (2.2)	46 (2.3)	0.7
PVD	88 (3.5)	89 (3.5)	0.2	72 (3.6)	68 (3.4)	−1.1
IHD	321 (12.6)	314 (12.3)	−0.8	231 (11.6)	229 (11.5)	−0.3

Abbreviations: BMI, body mass index (calculated as weight in kilograms divided by height in meters squared); CCI, Charlson comorbidity index; CHF, congestive heart failure; COPD, chronic obstructive pulmonary disease; C-SNP, chronic condition special needs plan; HHD, home hemodialysis; ICHD, in-center hemodialysis; IHD, ischemic heart disease; NOC, nocturnal dialysis; NS, not shown to protect patient privacy; PD, peritoneal dialysis; PVD, peripheral vascular disease; SNP, special needs plan.

^a^ C-SNP enrollees and matched patients were exactly matched on this factor.

In the county-matched analysis, 1986 C-SNP enrollees were matched to patients, balancing the covariates between the 2 groups (mean (SD) age, 57.8 [12.2] years vs 58.1 [12.2] years; 705 [35.5%] women in both groups). The same trends were noted in terms of distribution based upon race (472 [23.8%] African American individuals in both groups) and ethnicity (1085 [54.6%] Hispanic individuals in both groups). Compared with patients in the facility-matched analysis, the C-SNP enrollees and matched patients in the county-level analysis contained slightly more individuals with diabetes as ESKD etiology and a slightly lower dialysis vintage (Table 1).

In both analyses, most patients underwent in-center hemodialysis. In both analyses, C-SNP enrollees and matched patients had no significant differences in frequent comorbid cardiovascular, respiratory, or vascular conditions (Table 1).

### Hospitalizations

**Figure 1.**
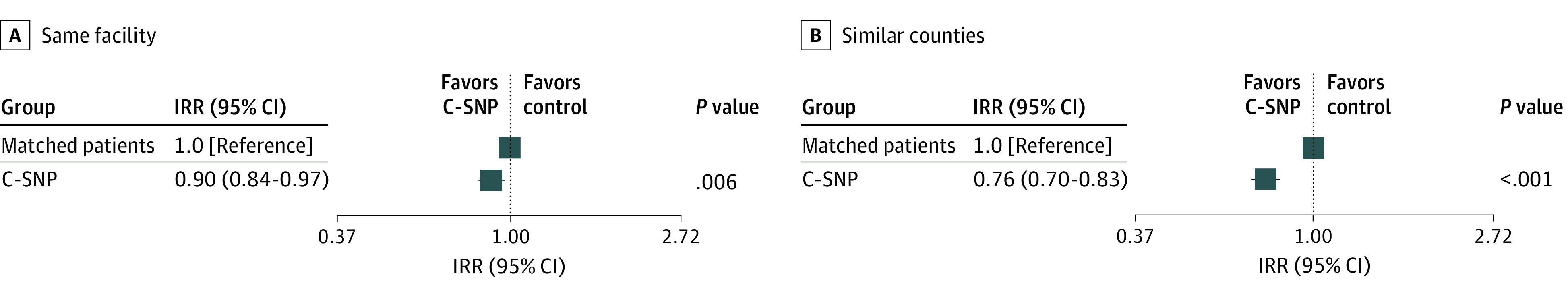
Hospitalizations Among Chronic Condition Special Needs Plan (C-SNP) Enrollees and Matched Patients Across Same Facility and Similar Counties IRR indicates incidence rate ratio.

In the facility-matched analysis, C-SNP enrollees experienced a total of 6141 hospital admissions during 55 561 patient-months of follow-up, corresponding to a hospitalization rate of 11.05 per 100 patient-months. Matched patients experienced a total of 6551 hospital admissions in 53 408 patient-months of follow-up, for a hospitalization rate of 12.27 per 100 patient-months. The incidence rate ratio for hospitalization was 0.90 (95% CI, 0.84-0.97; *P* = .006), favoring C-SNP enrollment (Figure 1A).

In the county-matched analysis, C-SNP patients experienced 4625 admissions in 44 553 patient-months of follow-up (rate, 10.38 per 100 patient-months). Matched patients experienced 6348 admissions in 46 704 patient-months of follow-up (rate, 13.59 per 100 patient-months). The corresponding incidence rate ratio was 0.76 (95% CI, 0.70-0.83; *P* < .001), favoring C-SNPs (Figure 1B).

### Mortality

**Figure 2.**
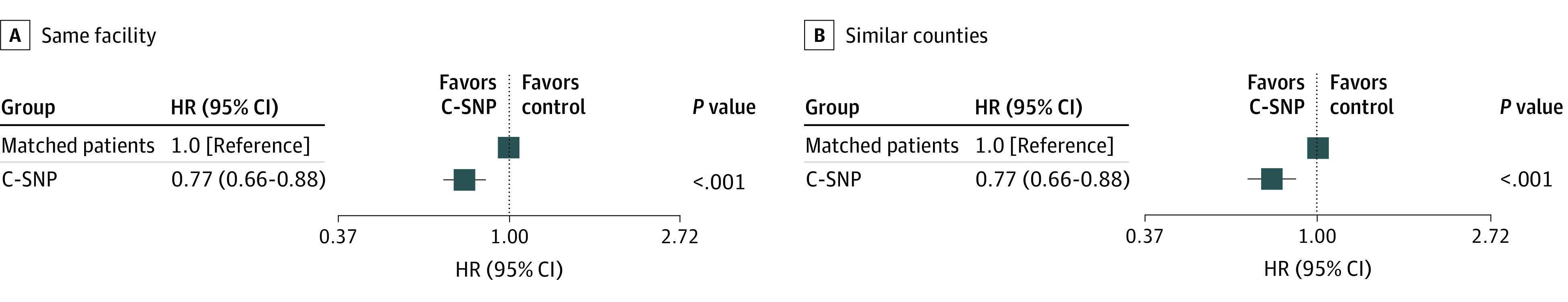
Mortality Among Chronic Condition Special Needs Plan (C-SNP) Enrollees and Matched Patients Across Same Facility and Similar Counties HR indicates hazard ratio.

In the facility-matched analysis, C-SNP enrollees experienced a total of 440 deaths during 55 561 patient-months of follow-up, corresponding to a mortality rate of 0.79 per 100 patient months. Matched patients experienced a total of 543 deaths during 53 408 patient-months of follow-up, for a mortality rate of 1.02 per 100 patient-months. Thus, C-SNP enrollees had a significantly lower HR for mortality compared with controls in the same facilities (0.77; 95% CI, 0.68-0.88; *P* < .001) (Figure 2A).

In the county-matched analysis, C-SNP enrollees experienced 337 deaths total during 44 553 patient-months of follow-up (mortality rate, 0.76 per 100 patient-months). Matched patients experienced a total of 461 deaths during 46 704 patient-months of follow-up (mortality rate, 0.99 per 100 patient-months). Thus, C-SNP enrollees had a significantly lower HR for mortality compared with controls in the same counties (0.77; 95% CI, 0.66-0.88; *P* < .001) (Figure 2B).

### Laboratory Values Indicative of Metabolic Control

No significant differences in calcium, potassium, or parathyroid hormone levels were observed between C-SNP enrollees and matched patients in the same facilities. A small but significant difference in phosphate levels was found, with C-SNP enrollees having lower mean (95% CI) phosphate levels than matched patients (5.4 [5.3-5.4] mg/dL vs 5.5 [5.4-5.5] mg/dL; *P* = .04) (Table 2). No significant differences in any laboratory parameters were observed between C-SNPs enrollees and matched patients in the same counties (Table 2).

**Table 2.** Metabolic Parameters Among C-SNP Enrollees and Matched Patients Across Same Facility and Similar County Analyses

Metabolic parameter	Patients in same facility			Patients in similar counties		
	Mean (95% CI)		*P* value	Mean (95% CI)		*P* value
	Matched	C-SNP		Matched	C-SNP	
Calcium, mg/dL	8.9 (8.8-8.9)	8.9 (8.8-8.9)	.67	8.9 (8.8-8.9)	8.9 (8.8-8.9)	.57
Phosphate, mg/dL	5.4 (5.3-5.4)	5.5 (5.4-5.5)	.04	5.4 (5.3-5.4)	5.4 (5.4-5.5)	.07
Potassium, mEq/L	4.9 (4.8-4.9)	4.9 (4.8-4.9)	.33	4.9 (4.8-4.9)	4.9 (4.8-4.9)	.83
PTH, pg/dL	521 (509-533)	523 (510-535)	.85	520 (507-534)	508 (495-522)	.22

Abbreviations: PTH, parathyroid hormone; C-SNP, chronic condition special needs plan.

SI conversion factors: To convert calcium to millimoles per liter, multiply by 0.25; potassium to millimoles per liter, multiply by 1.0; and PTH to nanograms per liter, multiply by 1.

### KDQOL-36 Survey Component Scores

**Figure 3.**
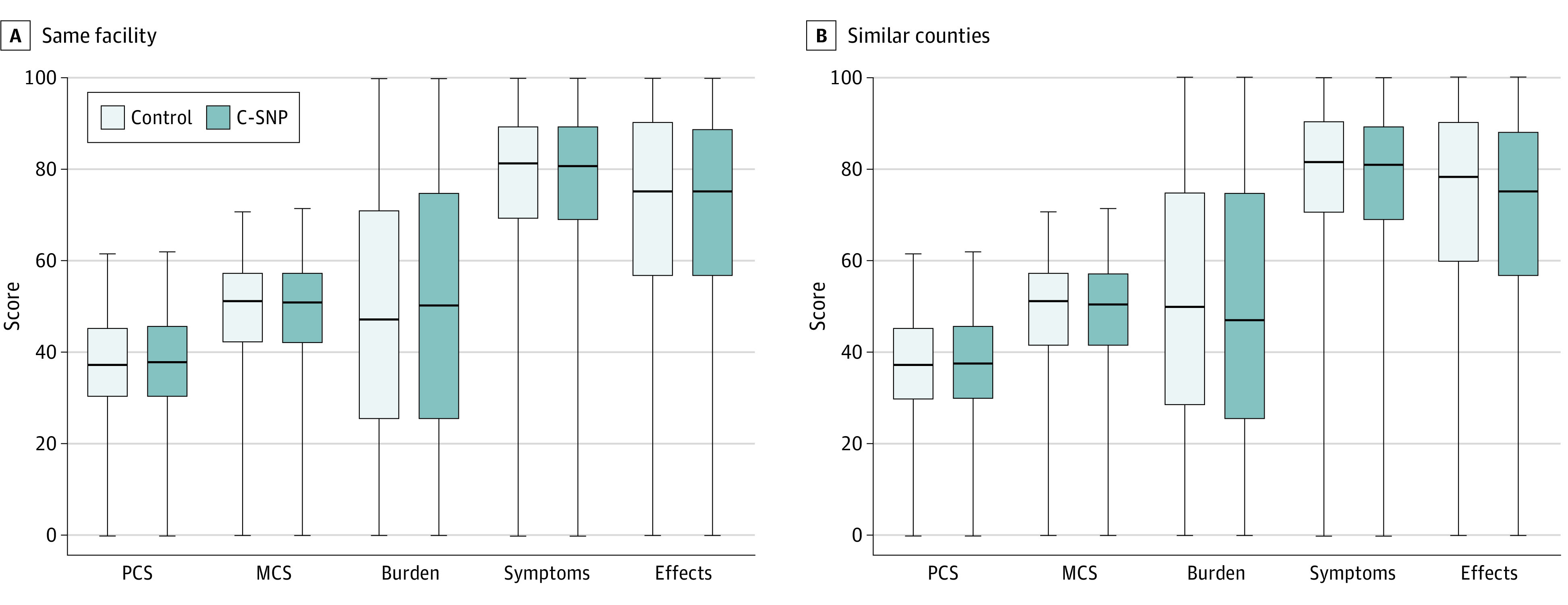
Kidney Disease Quality of Life Scores Among Chronic Condition Special Needs Plan (C-SNP) Enrollees and Matched Patients Across Same Facility and Similar Counties MCS indicates mental component score; PCS, physical component score.

No significant differences in KDQOL-36 scores were observed for C-SNPs enrollees compared with matched patients in the same facilities or in similar counties (Figure 3). The absence of any significant differences between groups was consistent across all of the domains assessed by the KDQOL-36 survey.

## Discussion

This analysis focused on determining whether C-SNP enrollment for patients with ESKD was associated with differences in outcomes compared with matched patients receiving care in the same facilities or in similar counties. Patients not enrolled in C-SNP were matched to C-SNP enrollees on the basis of a number of parameters and a propensity score that included multiple clinical and demographic variables. C-SNP enrollees had a lower rate of hospitalization and a decreased likelihood of mortality compared with matched patients in the same facilities and similar counties. No material differences in metabolic laboratory values or quality-of-life indices were observed between C-SNP enrollees and matched patients.

Although no prior examinations of the association of C-SNP enrollment with outcomes among patients with ESKD have been published, a few studies have highlighted the benefits of C-SNPs in other chronic diseases. In 2012, Cohen and colleagues^8^ reported the consequences the care model had for the Care Improvement Plus plan. They demonstrated that diabetic patients enrolled in the C-SNP had lower rates of hospitalization and readmission compared with fee-for-service Medicare beneficiaries in states where the plan operated. The care model, which included house calls, nurse care managers, pharmacist assistance to reduce medication-related problems, social service support, and support in transitions of care and end-of-life assistance, was associated with a greater reduction in hospital days for beneficiaries who were not White individuals (27% lower) with a concomitant increase in physician office visits (26% greater).

Avalere Health published a white paper that also demonstrated the benefits of C-SNPs for diabetic patients.^9^ In their analysis, C-SNP enrollees were 38% less likely to be hospitalized compared with non–C-SNP Medicare Advantage members, 32% less likely to be readmitted to hospital after a previous admission, and more likely to have primary care physician visits, have appropriate diagnostic testing, and have antidiabetic prescriptions filled. Approximately 15% of the C-SNP enrollees had chronic kidney disease or ESKD, compared with nearly 11% of non–C-SNP enrollees in this analysis.

The care model for C-SNP patients in this study has been described previously^10^ and is structurally similar to the Care Improvement Plus model. Care managers provided patient education and preventive care while using an integrated care management information system to identify and manage comorbidities and additional patient needs. Care managers also collaborated with the interdisciplinary dialysis team, including the patient’s nephrologist, who treats the patient’s ESKD, introducing or reinforcing patient-centered actions. In addition, the care managers focused on care transitions to minimize errors, address medication management, coordinate follow-up care, and facilitate access to patients’ specific benefits when possible.

To our knowledge, our analysis is the first to focus on C-SNPs for patients with ESKD. Although retrospective, it used a unique matching strategy to determine whether C-SNP enrollees had different outcomes than patients not enrolled in C-SNPs. The validity of these analyses is predicated on controlling confounding. To minimize confounding based on measurable factors, we used aggressive matching, in which C-SNP patients and non–C-SNP patients were exactly matched on several key potential confounders and by propensity score that included many other potential confounders. Moreover, we used strategies aimed at mitigating unmeasured confounding.

In the first analysis, we selected matched patients who received dialysis in the same facilities as their C-SNP counterparts to increase the likelihood that the 2 groups would be similar in terms of environmental factors that can influence health outcomes (eg, economic status, food availability, social cohesion). However, this approach limited matched patients to those who did not participate in C-SNPs despite having been eligible and having the opportunity available. Consequently, results were potentially subject to health participant bias. Therefore, we undertook the second analysis, in which matched patients were selected from similar counties (based on social, economic, and health-related factors) but had no C-SNP available. Such patients were likely to have similar environments as their C-SNP counterparts and may have participated in a C-SNP had it been available.

Interestingly, there were no significant differences in KDQOL-36 domain scores between the 2 groups in either analysis. The KDQOL-36 is a 36-question survey assessing patient self-perception of physical and mental functioning, burden of kidney disease, general health-related symptoms and problems, and the effects of kidney disease on daily life and activities. While many patients with ESKD complete the KDQOL-36 survey when requested, KDQOL data were incomplete and collected at random times throughout the study; thus, patient health perceptions may not be associated with component scores, and KDQOL-36 scores may be limited in detecting a difference from baseline. This means that the KDQOL-36 survey might potentially miss factors that are important to patients regarding their health.^11^ In this context, the perceived value of C-SNP enrollment, the benefits that patients can access, and the consequences of the care model might be missed by the KDQOL-36 survey content. Finally, C-SNPs are required to survey patients regarding patient experience,^12^ but such surveys do not focus on quality of life, and in this study, similar surveys were not available for controls.

Uniquely, in this study, Hispanic patients represented the largest proportions of study cohorts due, in part, to the geography of the C-SNPs studied. Hispanic individuals have a higher ESKD prevalence than non-Hispanic White individuals,^13^ and the disease process differs from non-Hispanic White patients in having a lower mortality risk, even accounting for diabetic status.^14,15^ Acknowledging the prevalence of Hispanics and African Americans in this study, Cohen et al^8^ identified an apparent benefit of C-SNP enrollment for individuals from minority racial and ethnic groups. In combination, these data suggest that C-SNP enrollment may provide greater access to care for patients from minority groups with downstream effects on patient health and health care utilization. Additional studies are needed to validate this hypothesis.

### Limitations

This study is subject to all limitations that affect retrospective analyses. This study is potentially confounded by the populations, given that patients who were eligible for C-SNP enrollment but declined may be fundamentally different than C-SNP enrollees. Facility-level differences in care and care team member complement may also be possible confounding factors. Additionally, accounting for potential shifts in physician coverage of patients and hospital programs is difficult during the study’s timeframe. Furthermore, the study population was concentrated in select geographies, and the results may not be applicable beyond these geographies.

## Conclusions

The data in this analysis suggested that enrollment in a C-SNP was associated with lower rates of hospitalization and mortality compared with similar patients who received ESKD care within the same facilities or within the same geographies but were not enrolled in C-SNPs. This suggests that aspects of the care model, including access to the integrated care team, regular interactions between this team and the interdisciplinary dialysis team (eg, the patient’s nephrologist), and possibly access to the additional services and benefits provided via C-SNPs, may improve patient outcomes beyond the standard of care for this high-risk, high-need population.

## Notes

Supplement. **eAppendix. **Additional Methods **eFigure. **Distribution of Propensity Scores Before and After Matching **eTable 1. **Censoring Reasons by Patient Type and Study Design **eTable 2. **Characteristics of Unmatched Patients
