# Supplementary material for: Association of Chronic Condition Special Needs Plan With Hospitalization and Mortality Among Patients With End-Stage Kidney Disease
Source: JAMA Netw Open. 2020 Nov 2;3(11):e2023663. doi: 10.1001/jamanetworkopen.2020.23663 (PMC7607441; doi:10.1001/jamanetworkopen.2020.23663)
Supplement: Supplement. — eAppendix. Additional Methods eFigure. Distribution of Propensity Scores Before and After Matching eTable 1. Censoring Reasons by Patient Type and Study Design eTable 2. Characteristics of Unmatched Patients [file jamanetwopen-e2023663-s001.pdf]

Supplemental Online Content

Becker BN, Luo J, Gray KS, et al. Association of chronic condition special needs plan with hospitalization and mortality among patients with end-stage kidney disease. *JAMA Netw Open*. 2020;3(11):e2023663. doi:10.1001/jamanetworkopen.2020.23663

eAppendix. Additional Methods

eFigure. Distribution of Propensity Scores Before and After Matching

eTable 1. Censoring Reasons by Patient Type and Study Design

eTable 2. Characteristics of Unmatched Patients

This supplemental material has been provided by the authors to give readers additional information about their work.

© 2020 Becker BN et al. *JAMA Network Open.*

eAppendix. Additional Methods

Data Source and Company Information

DaVita Kidney Care treats more than 200,000 dialysis-dependent patients at more than 2,700 outpatient dialysis centers in the United States and presently has more than 4,000 patients receiving care via a C-SNP. Patient-level data are routinely recorded for all patients in an electronic health record system and are available in a de-identified format for research purposes.

CareMore, a subsidiary of Anthem Inc., is an integrated health plan and care delivery system for Medicare and Medicaid patients, delivering care to chronically ill patients through its C SNPs. It currently operates in 9 states and Washington, D.C.

Control Matching

Missing values were imputed via a tree-based method. Inputs were then preselected after standardization before conducting cluster analyses. The pre-specified number of clusters ranged from 14 to 15 for each of the index years.

© 2020 Becker BN et al. *JAMA Network Open.*

eFigure. Distribution of Propensity Scores Before and After Matching

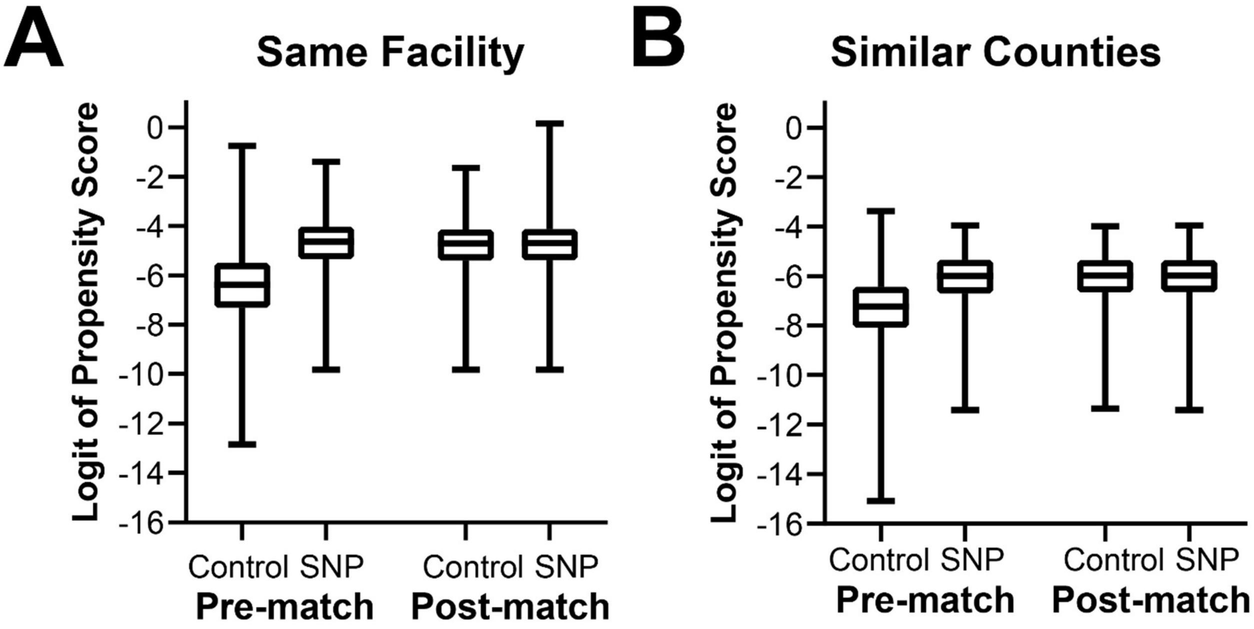

The minimum, 25th percentile, median, 75th percentile, and maximum values for the logit of the propensity score used in matching are presented for control and SNP patients before and after matching for the matching strategy involving patients in the same facility (A) and the strategy involving similar counties (B).

© 2020 Becker BN et al. *JAMA Network Open.*

eTable 1. Censoring Reasons by Patient Type and Study Design

	Censoring Reason		
	**End of study**	**Death**	**Other [a]**
**Same facility**			
Control, n (%)	1175 (46.2)	543 (21.3)	827 (32.5)
SNP, n (%)	1268 (49.8)	440 (17.3)	837 (32.9)
**Similar counties**			
Control, n (%)	1033 (52.0)	461 (23.2)	492 (24.8)
SNP, n (%)	1016 (51.2)	337 (17.0)	633 (31.9)

[a] Includes unenrollment from SNP (cases), insurance change (controls), recovery, transfer, withdrawal, and transplant
Abbreviations: SNP, special needs program

© 2020 Becker BN et al. *JAMA Network Open.*

eTable 2. Characteristics of Unmatched Patients

	Same Facility (N=28,302)	Similar Counties (N=108,051)
**Age**, years, mean ± SD	60.9 ± 14.8	59.2 ± 14.6
**Sex**, female, n (%)	13,010 (46.0)	49,868 (46.2)
**Race**, n (%)		
White	5135 (18.1)	28,063 (26.0)
Black	6848 (24.2)	42,164 (39.0)
Hispanic	11,985 (42.4)	25,609 (23.7)
Asian	2540 (9.0)	6001 (5.6)
Other/missing	1794 (6.3)	6214 (5.8)
**CCI**, mean ± SD	5.5 ± 1.9	5.3 ± 1.9
**Diabetes**, n (%)	21,954 (77.6)	78,866 (73.0)
**Modality**, n (%)		
HHD	31 (0.11)	1233 (1.1)
ICHD	26,149 (92.4)	97,545 (90.3)
NOC	176 (0.6)	681 (0.6)
PD	1946 (6.9)	8592 (8.0)

Abbreviations: CCI, Charlson comorbidity index; HHD, home hemodialysis; ICHD, in-center hemodialysis; NOC, nocturnal dialysis; PD, peritoneal dialysis; SD, standard deviation.

© 2020 Becker BN et al. *JAMA Network Open.*
